# How personality functioning relates to psychological distress and behavioral attitudes during the Covid-19 pandemic

**DOI:** 10.1007/s00406-023-01722-7

**Published:** 2024-01-06

**Authors:** Leonie Kampe, Susanne Hörz-Sagstetter, Johannes Bohn, Carina Remmers

**Affiliations:** 1 Department of Psychological Diagnostics, International Psychoanalytic University Berlin, Stromstrasse 1, 10555 Berlin, Germany; 2 Department of Clinical Psychology and Psychotherapy, Psychologische Hochschule Berlin, Berlin, Germany; 3 Department of Education and Psychology, Division of Clinical Psychological Intervention, Freie Universität Berlin, Berlin, Germany; 4 Department of Psychology, Institute for Mental Health and Behavioral Medicine, HMU Health and Medical University, Potsdam, Germany

**Keywords:** Personality functioning, Social distancing, Defense mechanisms, Narcissism, COVID-19, Psychological distress

## Abstract

Functional aspects of personality are crucial for experiencing and handling emotional distress. With the outbreak of the Covid-19 virus and the subsequent installation of mitigation rules of social distancing, severe psycho-social challenges were posed upon people. Research has shown that individuals react differently to these challenges. This study aimed to investigate the role of dimensional aspects of personality during the Covid-19 pandemic. Specifically, we examined how personality functioning, defense mechanisms, and narcissism were related to psychological distress and cognitive and behavioral attitudes towards the rules of social distancing. In a non-clinical sample (N = 254), Level of Personality Functioning Scale, Inventory of Personality Organization, Defense Style Questionnaire, Pathological Narcissism Inventory, and three single questions regarding emotional distress and behavioral attitudes towards the pandemic were used. Structural equation models with reference and residual factors were calculated. Impairments in personality functioning and vulnerable narcissism showed significant positive relationships, adaptive defense mechanisms significant negative relationships with psychological distress during the pandemic. Residual factors for aggression and low moral values showed distinct negative relationships with psychological distress related to social distancing. Among individuals who chose to ignore the rules of social distancing, greater impairment in personality organization was found. Personality functioning may elucidate individual differences in psychological distress and compliance with the mitigation rules during the pandemic. Limitations of measures are carefully considered in all interpretations.

## Introduction

How individuals deal with stressful situations depends on different aspects of their personality [1–4]. With the outbreak of Covid-19, a global health threat posed many challenges to the public: Not only did the unpredictable health threat itself cause existential anxieties and emotional distress, but the resulting installation of the restricting measures of social distancing also led to severe psycho-social consequences [5–10]. By now, many studies have demonstrated the immense impact of the pandemic and the mitigation measures on people’s mental states [5, 11–15]. However, psychological adaptation to the pandemic and willingness to comply with the restricting measures do not merely depend on external factors [16–18]: For example, studies demonstrated that openness and extraversion acted as resilience factors [1, 2, 19, 20] whereas neuroticism could be seen as a vulnerability factor for experiencing emotional distress during the pandemic [3, 21–26]. Targeting the important question of protective factors in these challenging times [27, 28], further studies have found that defense mechanisms [21, 29–31], coping strategies, and resilience [32–38] were significantly related to distress during the pandemic. Regarding behavioral aspects such as compliance with the rules of social distancing, studies have focused on how different reactions and attitudes are related to egocentric, unempathetic, and antisocial traits, but come to dissenting conclusions [39–42].

Consequently, different aspects of personality have shown to be relevant for understanding an individual’s emotional, cognitive, and behavioral reactions towards the pandemic and the related mitigation rules [43]. For that reason, we aimed to investigate further personality aspects, specifically dimensions of personality functioning, and their relationships with reactions towards the pandemic.

### Personality functioning

With the introduction of the Level of Personality Functioning Scale (LPFS; Criterion A) [44] of the Alternative Model of Personality Disorders (AMPD) in DSM-5 [45] and the dimensional approach to diagnosing personality disorders in ICD-11 [46], functional aspects of personality have received interest for understanding and diagnosing personality pathology. Extensive empirical research on the LPFS [47] has shown that more impairment in personality functioning (PF) is related to lower abilities of coping with stress, more need for psychiatric hospitalization, and overall more problems with mental health [48–50]. Furthermore, impairment in PF is related to antisocial behavior [51, 52], which is associated with less compliance with the mitigation rules during the pandemic [53]. It can be assumed that a lack of compliance can also be understood as a consequence of reduced internal capacities to regulate personal drives and egocentric wishes, as well as considering other people’s needs in reciprocal relationships. To date there are only few studies focusing on the question if psychological distress during the Covid-19 pandemic and certain cognitive and behavioral reactions regarding the rules of social distancing are related to dimensions of PF [54].

For that reason, the first aim of our study was to investigate how impairments of personality capacities are related to emotional distress and cognitive and behavioral attitudes during the pandemic. For the assessment of personality impairment, we chose two different dimensional measures which were (a) based on the recently introduced framework of the AMPD in DSM-5, operationalized by the LPFS, and (b) based on the psychodynamic concept of personality organization (PO; [55]). The model of PO also assesses personality impairment through a dimensional model but derives its dimensions from the psychodynamic background of object relations theory. The domains are identity, quality of object relations, defense mechanisms, aggression, moral values, and reality testing. Although the two approaches differ to some extent, they show a large conceptual overlap [48, 49]. There is increasing consensus that functional impairments in personality represent a general factor of psychopathology [56]. Our interest lays in how PF in general and its dimensions are related to dealing with the pandemic and the restrictions of social distancing. Among other questions we were specifically interested if impairments in the integration of moral values are related to less compliance with the rules of social distancing.

### Defense mechanisms

Defense mechanisms are defined as automatized psychological reactions to keep unpleasant affects, conflicts or fears out of awareness. Defense mechanisms can be spanned on a continuum from adaptive, neurotic, to maladaptive mechanisms [57]. Studies have shown that the dominant use of adaptive defense mechanisms is linked to mental health, especially during emotional distress [58–61]. It has also become apparent that less adaptive mechanisms are related to more impairment in PF and personality disorders in general [62, 63]. Some studies set the stage for investigating the role of defense mechanisms during the pandemic, but mainly focused on their relationship with emotional coping [29, 31, 64, 65]. With our study, we aimed to further study the role of defense mechanisms and the experience of emotional distress specifically related to the challenges of the pandemic, and furthermore wanted to investigate their relationship with cognitive and behavioral attitudes towards the rules of social distancing.

### Narcissism

Our third aim was to explore the character trait of narcissism and how it is related to emotional, cognitive, and behavioral reactions to the pandemic. The concept of narcissism has been under discussion, leading to the conclusion that narcissism is a construct with many facets [66, 67]: Studies have shown that there is a grandiose facet of narcissism, and a vulnerable side to it [68–72]. Grandiose narcissism describes the facet of arrogant behavior, sense of entitlement, a derogating attitude towards others, and an inflated self-esteem. Vulnerable narcissism is understood as an underlying facet of insecurity with low self-esteem, high sensitivity, and entitlement rage [73]. While grandiose narcissism is related to better coping in emotional distress and overall better psycho-social functioning, including the use of more adaptive defense mechanisms, vulnerable narcissism is related to psychopathology, low abilities of stress coping, and a dominant use of maladaptive defense mechanisms [59, 70, 74–78]. Furthermore, grandiose and vulnerable narcissism are differently related to pro-social behavior and antisocial tendencies [53, 79, 80].

Consequently, the two facets of narcissism are ambiguously related to emotional coping abilities and behavioral attitudes towards other people. For that reason, we wanted to explore how dimensional expressions of grandiose and vulnerable narcissism are related to emotional and behavioral reactions during the pandemic and the rules of social distancing. In dealing with the restrictions, it could be that individuals with high levels of narcissism experienced them as an insult, hindering them in fulfilling their narcissistic needs and therefore reacted strongly.

### Research questions and hypotheses

Our study aimed to investigate the relationship between dimensional aspects of personality (PF, PO, defense mechanisms, and narcissism) and emotional, cognitive, and behavioral reactions during the Covid-19 pandemic and the related rules of social distancing. We differentiated our research questions accordingly into three different areas: (a) the overall reactions towards the health threat of the pandemic, (b) the reactions specifically towards the consequences of social isolation, and (c) the willingness to comply with the rules of social distancing. From our point of view, a differentiation between these three areas is of interest because they each address different aspects of PF such as emotion regulation abilities, capacity for being alone and independent, as well as interpersonal abilities such as empathy and the ability to consider other people’s needs as equally important as one’s own desires. Our three main hypotheses derived from the theorizing outlined above were preregistered at Open Science Framework (OSF; https://osf.io/9tuqd) prior to the data analysis.^1^ Our fourth research question was explorative and had not been preregistered:

#### ***Hypothesis 1.***

Higher levels of PF (less impairment in PF and PO) relate negatively to the experience of psychological distress caused by the restrictions of the Covid-19 pandemic.

#### ***Hypothesis 2.***

The use of adaptive defense mechanisms (DSQ-40) relates negatively to the experience of psychological distress caused by the restrictions of the Covid19 pandemic.

#### ***Hypothesis 3.***

Higher levels of impairment in Moral Values (IPO-30) relate negatively to compliance with the rules of social distancing.

#### ***Exploratory research question (H4).***

How are grandiose and vulnerable aspects of narcissism related to emotional distress and cognitive and behavioral attitudes towards the rules of social distancing?

## Materials and methods

The study procedure was preregistered in full detail at Open Science Framework (OSF; https://osf.io/9tuqd/) and was administered through an online survey as part of a larger study. IRB approval was obtained from the IRB committee of Psychologische Hochschule Berlin prior to data collection. Data collection took place between September and November 2020 in Germany.^2^ A non-clinical sample of N = 254^3^ [192 females, 59 males, and 3 diverse]^4^ was recruited via mailing lists. Inclusion criteria were a minimum age of 18 years and sufficient German language skills.

### Measures

We assessed emotional, cognitive, and behavioral reactions to the pandemic by the following three items specifically designed to address the unprecedented situation of the pandemic and restrictions:

*Psychological distress due to the challenges of the pandemic in general:* “Please rate your subjective psychological distress due to the circumstances and consequences of the Covid-19 pandemic from approximately March 2020 until today” on a rating scale from 1 (*no distress*) to 5 (*severe distress*).

*Psychological distress due to the rules of social distancing:* “Please estimate your subjective psychological distress due to the rules of social distancing since the outbreak of the Covid-19 pandemic and the implementation of the restrictive measures” on a rating scale from 1 (*no distress*) to 3 (*constant distress*).

**Fig. 1:**
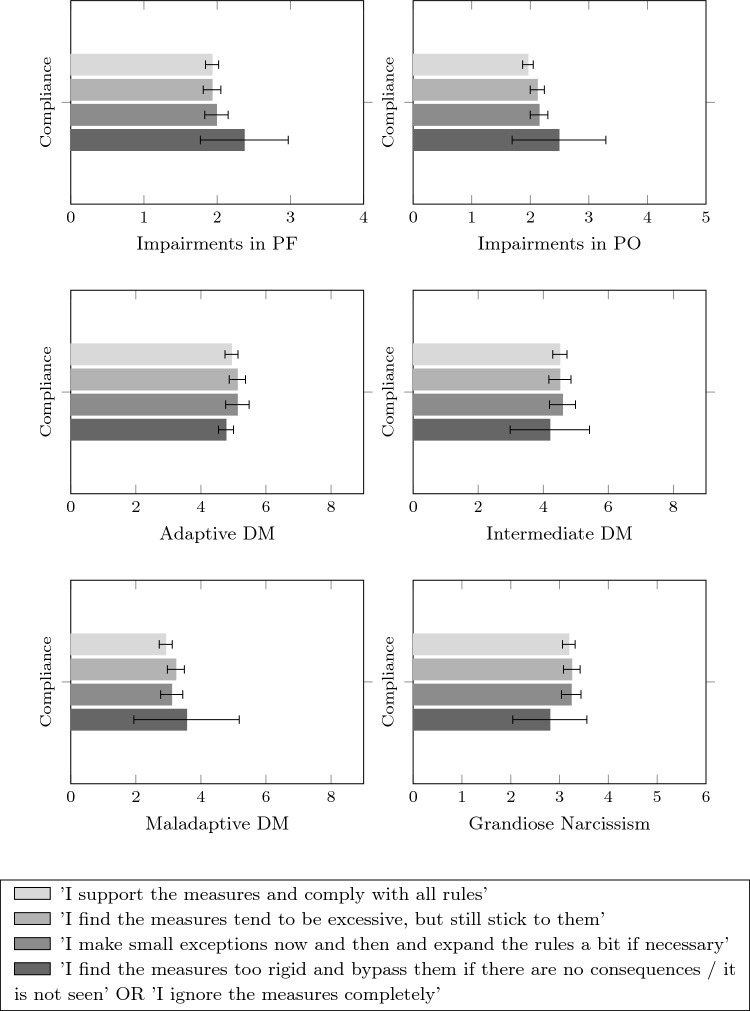
Means of different personality dimensions for the four compliance groups

*Cognitive and behavioral attitudes towards the rules of social distancing:* “Please describe how consistently you have been following the regulations and rules regarding social distancing since the outbreak of the Covid-19 pandemic and the start of the restrictions (approx. March 2020—today) and what your attitude has been towards them” on a rating scale from 1 (*highly compliant*) to 4 (*ignoring the rules*). With this item, we tried to carefully differentiate between a critical attitude towards the rules of social distancing and the choice to completely ignore them. Therefore, the rating scale of this item was not strictly ordinal, all four answer options are displayed in Fig. 1 and in the Appendix. Since the different response categories cannot be placed in a clear order, this item was treated as a nominally scaled variable in all analyses.

*Psychological Distress.* To test the validity of our items on psychological distress during the pandemic, we assessed general psychological distress with the Brief Symptom Inventory [81]. Items are rated on a 5-point Likert scale ranging from 0 (*not at all*) to 4 (*extremely*). To obtain an overall score for psychological distress, the Global Severity Index (GSI, Cronbach’s α = 0.93 in this data set) is calculated from the three subscales.

*Personality Functioning.* PF was assessed with the German 12-item version [82] of the DSM-5 LPFS-BF [83], capturing the four LPFS domains identity, self-direction, empathy and intimacy [45]. The items are rated on a 4-point Likert scale, ranging from 0 (*totally false*) to 3 (*very true*). Higher scores display greater impairment in PF. The LPFS-BF showed good psychometric properties with α = 0.86 in this data set.

*Personality Organization.* The 30-item version of the Inventory of Personality Organization [84] was employed to assess PO [85]. Items are rated on a 5-point Likert scale from 1 (*never true*) to 5 (*always true*). The IPO-30 scores follow a bifactor model with one general factor (PO) and three specific factors (aggression, moral values, reality testing). Higher scores display greater impairment in PO. The IPO-30 showed acceptable psychometric properties with α = 0.79 for the general factor, α = 0.79 for the specific factor of aggression, α = 0.76 for the specific factor of moral values, and α = 0.80 for the specific factor of reality testing in this data set.

*Defense Mechanisms.* We used the German 40-item version of the Defense Style Questionnaire [86, 87]. The DSQ-40 measures 20 defense mechanisms with two items each, rated on a 9-point Likert scale ranging from 1 (*not at all*) to 9 (*absolutely*). For the assignment of the items to the scales adaptive, neurotic and maladaptive mechanisms see [59]. While the psychometric properties were good for the maladaptive mechanisms (α = 0.88), they were only questionable for the adaptive mechanisms (α = 0.65), and only poor for the intermediate mechanisms (α = 0.56) in this sample.

*Narcissism.* We used the German version of the Pathological Narcissism Inventory [69, 72, 88]. The PNI is a measure for grandiose and vulnerable features of narcissism and contains 54 items, scaled across the seven subscales: exploitativeness, grandiose fantasy, self-sacrificing self-enhancement, entitlement rage, devaluing, contingent self-esteem, and hiding the self. Items are rated on a 6-point Likert scale ranging from 0 (*not at all like me*) to 5 (*very much like me*). Our analysis is based on a bifactor solution (for the assignments of the scales see [59]). The psychometric properties were good for grandiose narcissism (α = 0.89) and even better for vulnerable narcissism (α = 0.94).

### Statistical analyses

To examine how PF, PO, defense mechanisms, and narcissism were related to psychological distress due to the pandemic, we used a total of six structural equation models. Structural equation models allow to reduce the influence of measurement error on the results. In each model, the corresponding factor (with indicators comprised of parcels) served as predictor of the two questions on distress (both of which were categorical variables).

In case of the models on narcissism and PO, S-1 models were used [89] to capture the bifactor structure. In these models, one facet was used as a reference, and the other facet (of narcissism or PO) was included in the model as residual factor, respectively. The residual factor then described the extent to which the values on this facet deviated from the reference facet. The reference and the residual factor were uncorrelated because the residual factor comprised those parts in the measurement error free variance of the residual facet that could not be predicted by the reference factor. In case of narcissism, grandiose narcissism was used as reference factor. Therefore, the residual factor for vulnerable narcissism represented those elements of vulnerable narcissism that were not predicted by grandiose narcissism. In case of PO, general impairment of PO was used as the reference factor. The different residual factors represented those parts within the specific facets that were not represented by general PO.

In the analyses of narcissism and PO, the factors of the S-1 model were used to predict distress due to the pandemic. According to this design, the coefficient for the regression of distress on the residual factors represented the influence of the residual factor on distress beyond the influence of the reference factor. Similar designs were already used in studies of narcissism [59] and other multi-facet constructs (e.g., [90–92]).

In the models for PF and defense mechanisms, there was only one factor predicting distress. We had no clear hypothesis regarding the direction of the relationships between general psychological distress and Covid-19 related psychological distress. Therefore, the associations between the different kinds of distress were examined in another structural equation model with undirected associations.

All structural models were estimated with MPlus 8.6 [93] using the weighted least square mean and variance adjusted estimator. We used the χ^2^-test, the CFI and the RMSEA to examine the goodness-of-fit. A non-significant χ^2^-test (or at least a value of χ^2^ < 2 * df), a CFI > 0.97, and a RMSEA < 0.05 are signs of a good model fit, values of χ^2^ < 3 * df, CFI > 0.95, and RMSEA < 0.08 are signs of an acceptable model fit [94].

To explore how different dimensional aspects of personality are related to cognitive and behavioral attitudes towards the rules of social distancing, we took a different approach: As this item was not measured on an ordinal but a nominal scale, individuals were assigned to a group based on their response category (1, 2, 3, 4). Means and confidence intervals of each group’s expressions on the personality dimensions were calculated. Since this analysis was based merely on manifest variables, only the values of the reference facet were included in the analysis for PO and narcissism. ANOVAs were used to test for differences between the groups.

Given that testing H3 required a different model, the results related to H1 and H2 and the exploratory research question H4 are presented first, followed by those of the H3.

## Results

### Emotional distress during the pandemic

*Initial Analyses.* The model fit of all models was good in most cases and acceptable in some (see Table 3 in the Appendix). The means and standard deviations for all scales are displayed in Table 4 in the Appendix, no detectable gender differences were found. We first tested the validity of our distress questions by calculating correlations between psychological distress related to the challenges of the pandemic in general and distress related to social distancing, and the BSI. We found correlations ranging from *r* = 0.319 to *r* = 0.422, indicating a clear, yet medium association. The standardized regression coefficients of all models are displayed in Table 1.

**Table 1:** Standardized regression coefficients of the relationship between aspects of personality and emotional distress during the pandemic

Predictor	Psychological distress due to the challenges of the pandemic	Psychological distress due to the rules of social distancing
PF	0.28*	0.25*
PO (reference factor)	0.14*	0.13*
PO – Reality Testing (residual factor)	− 0.12	− 0.03
PO – Aggression (residual factor)	− 0.21*	− 0.19*
PO – Moral Values (residual factor)	− 0.09	− 0.25*
Adaptive defense mechanisms	− 0.27*	− 0.15
Intermediate defense mechanisms	0.04	0.16
Maladaptive defense mechanisms	0.09	0.10
Grandiose narcissism (reference factor)	0.00	0.02
Vulnerable narcissism (residual factor)	0.30*	0.26*

The models of PO and narcissism are S-1 models with a reference factor and a residual factor, which represents those parts of this specific facet not captured by the reference factor

*significant path coefficients

*Personality Functioning.* We found significant correlations between impairments in PF and psychological distress related to both, the pandemic in general and to social isolation: the stronger the impairment in PF, the more distress was reported.

*Personality Organization.* We found significant correlations between impairments in PO and psychological distress related to the pandemic in general and to social isolation. Together with the results on impairments in PF, this confirms H1. Furthermore, we found that the residual factor *aggression* showed significant negative correlations with both items of psychological distress: subjects with higher levels of aggression than expected based on the general level of PO reported lower psychological distress related to the challenges of the pandemic in general and to social distancing. A similar association was found for the residual factor *moral values:* subjects with higher impairment in moral values reported lower psychological distress related to the challenges of the pandemic in general and to social distancing.

*Defense mechanisms.* Higher usage of adaptive defense mechanisms was significantly negatively related to psychological distress due to the challenges of the pandemic in general, but not to psychological distress related to the rules of social distancing. Consequently, H2 was confirmed.

*Narcissism. Grandiose narcissism* was not significantly related to psychological distress during the pandemic. However, *vulnerable narcissism* showed a significant positive relationship with psychological distress related to both, the pandemic in general and social isolation. This answers our explorative research question (H4).

### Cognitive and behavioral attitudes towards the rules of social distancing

The results for the different cognitive and behavioral attitudes towards the rules of social distancing are displayed in Fig. 1 and in Table 5 in the Appendix. While there is a large overlap in the confidence intervals, there is a significant difference between the groups regarding the general factor of the PO with *F*(3,247) = 4.02, *p* = 0.008: People who reported to bypass the mitigation measures showed significant impairments in PO. The ANOVAs for all other variables (including the specific factor of moral values in the PO) were not significant. Consequently, H3 was not confirmed.

## Discussion

In this study, our goal was to investigate how dimensional aspects of personality were related to emotional, cognitive, and behavioral reactions to the Covid-19 pandemic. As many studies have examined the psychological burden and increase in mental disorders during the pandemic [7–9, 27–29, 95–99], we specifically aimed to study how core dimensions of personality are related to how individuals dealt with the pandemic. Our study has three main findings:

First, more impairment in PF was related to the experience of emotional distress during the pandemic and due to the rules of social distancing (H1). Along these lines, previous studies have shown that individuals with personality disorders were especially affected by the pandemic and the social isolation rules [98, 100]. By using the dimensional approach of measuring personality impairment according to current diagnostic models ICD-11 or DSM-5 AMPD, our study extends previous research and highlights the importance of basic capacities for understanding individual differences in how the pandemic affected well-being and psychological health. Functional aspects of personality have not only shown to be of predictive value for mental health [49] but also to provide specific implications for psychotherapy [56].

Paradoxically, the residual factors for impairments in the *regulation of aggression* and *integration of moral values* were significantly related to *lower* experience of emotional distress due to the rules of social distancing. One explanation could be that these dimensions are especially impaired in pathological narcissism [74, 76, 77, 101, 102], which is associated with tendencies to project fears onto other people or use denial and omnipotence [103] and thus functions as psychological defense. This also matches our findings of grandiose narcissism not being related to emotional distress during the pandemic (H4). Another explanation might be that high expressions of these traits have been found to be linked with a lack of compliance with the rules of social distancing [41] and therefore not related to emotional distress. This conclusion matches other studies showing that certain personality traits like boldness or neuroticism were associated with “dysfunctional” behavior during the pandemic [104, 105].

Even though causal conclusions should not be drawn from the current findings, a tentative interpretation could be that individuals who are less concerned about others and who show a more pronounced self-orientation experienced lower levels of distress during the pandemic, which is in line with previous findings on the defensive function of narcissistic traits [59]. This would support general considerations of trait-like factors such as narcissism as defensive operations, which should gain further consideration in research and theoretical understanding. In interpersonal personality models for example, personality is conceptualized as the result of dynamic processes [106].

Our second main finding shows that the adaptiveness of defense mechanisms was associated with less distress due to the pandemic and restrictions (H2). Hence, a more flexible way of regulating stressors may have been helpful in dealing with strains of the pandemic, which is in line with prior studies on the protective function of defense mechanisms during the pandemic [31]. This matches our findings of vulnerable narcissism being strongly related to the experience of emotional distress due to the pandemic (H4). As demonstrated in prior studies, unlike grandiose narcissism, people with vulnerable narcissism use less adaptive emotion regulation strategies [59]. Altogether, these findings furthermore strengthen the suggestion of taking underlying regulatory abilities more into account in diagnostic and psychotherapeutic settings [30].

Our third finding was that people who explicitly reported to bypass the rules of social distancing showed higher levels of impairment in PO. This relationship was found on the overall level of PO, but not on the level of less integrated moral values, where we had expected an association with less compliance (H3). Two possible explanations are the non-clinical composition of our sample as well as the limitations of the construction of our compliance measure. Thus, this question should be re-examined with clinical samples. Although the established association points toward the conclusion of reduced compliance being related to personality impairment, this finding needs to be interpreted carefully due to the single item construction and its two-fold meaning, including a question related to both, behavior and to a cognitive attitude. Here, validity and reliability problems are to be noted as well as limitations due to the self-report nature of the measures in general. Future studies may be well advised to use behavioral indicators to more thoroughly track how people’s actions relate to personality dimensions in situations of high stress. Overall, the current findings add to the results on less adherence to the rules of social distancing associated with certain personality traits like boldness, narcissistic rivalry, dark triad traits [39, 104, 107–110] or attachment styles. However, it is important to highlight that this study does not imply that a critical attitude towards the mitigation rules equals personality impairment, but amongst those who chose to ignore the rules, elevated personality impairment was found.

*Practical conclusions.* Taken together, our results are in line and extend previous research [3, 6, 21, 39, 111–113] on increased symptom load and the role of personality traits by tentatively shedding light on how individual differences in basic capacities of regulating the self and the relationship with others may explain both the experience of distress and also the way people think about and behave toward the rules that were initiated during to the pandemic [114]. A deeper understanding of these aspects could prospectively help to prevent risk groups from emotional decompensation [115], and to understand and address behavioral resistance towards the mitigation rules. Furthermore, if researchers and representatives of the public health field aim to change people’s attitudes and behaviors (e.g., compliance to mitigation rules), our results demonstrate that it is necessary to understand why people think and act the way they do. Our findings tentatively suggest that the latter may be associated with underlying regulatory capacities of personality, especially in situations of collective fear and global health threats. For example, a tendency toward grandiose narcissism may protect the individual from experiencing emotional distress (acting as a defense mechanism by providing a strong and potent self-representation) but may on the other hand lead to problematic behaviors and attitudes. Thus, an exploration of the underlying psychological mechanisms is important if one seeks to a) help people reduce emotional and psychological distress, and b) change attitudes and problematic behaviors.

*Limitations.* The conclusion of our findings should be interpreted carefully due to some considerable limitations: 1) Our question regarding cognitive and behavioral attitudes towards the rules of social distancing is restricted by some methodological limitations: 1) as a single item measure, this item was developed specifically for the unprecedented pandemic and has not been tested for validity. Also, its lack of psychometric scaling only allows qualitative interpretations of the groups. 2) Our two items on psychological distress are also single item measures. While these items have a clearly interpretable response scale, they are still newly designed and not established items. 3) This study consists of a non-clinical sample and needs to be replicated with a clinical sample, and 4) it does not provide longitudinal data to allow causal interpretations.

*Future research.* Future studies should use more robust scales to differentiate experienced distress from emotional, cognitive, and behavioral aspects regarding how people deal with distress, than realized in our study. In addition, experience sampling methods directly tapping into people’s daily life would help to investigate how dynamic processes of stress reaction and subsequent attitude formation and behavioral consequences depend on individual differences in PF [116]. Furthermore, future research should realize a broader assessment of maladaptive personality traits and psychopathological symptoms to follow up on emerging comprehensive models of psychopathology such as HiTOP [117]. The latter may elucidate the position that impairment in PF takes up in an empirically based and hierarchically organized model of psychopathology [118]. If PF was the strongest predictor of distress and dysfunctional attitudes and behavior, this would align with psychodynamic accounts where PF forms one of the core etiological concepts [119], herein called structural integration [120]. From a practical perspective, Bach and Simonsen [56] have recently outlined how impairment in PF may have important clinical implications. The latter should be considered in intervention programs for people who suffer severely and long-term from stressors such as the pandemic.

Our study bolsters the idea that PF may play a central role in understanding individual differences in emotional, cognitive, and behavioral reactions towards challenging situations. Although this study is a cross-sectional design with clear methodological shortcomings, our results may motivate further research on the role of strengthening regulatory abilities of personality as potential central factors for prevention of mental illness.

## Data Availability

The raw data supporting the conclusions of this article will be made available by the authors upon request.

## Appendix

Answer options for the Covid-19 related questions on psychological distress and cognitive and behavioral attitudes:

1. The answers for *psychological distress related to the general health threat of the pandemic* were combined in three ordinal categories, ranging from 1 (*no distress*) over 2 (*situational concern, but no ongoing psychological distress*) to 3 (*Constant psychological distress*).
2. When asked about the *psychological distress caused by social distancing*, individuals could respond on a scale of 1 (‘*no distress’*) to 5 (‘*mentally ill resulting in psychotherapeutic treatment*’), with higher scores representing greater distress. For the evaluation, the sparsely populated response categories 4 (‘*psychologically clearly and persistently burdened*’) and 5 were combined into one category.
3. For the third question, participants rated their compliance from 1 (‘*I support the measures and comply with all rules’*) over 2 *(‘I find the rules tend to be excessive, but still stick to them’*) and 3 (‘*I make small exceptions now and then and expand the rules a bit if necessary’*) to 4 (‘*I find the measures too rigid and bypass them if there are no consequences / it is not seen’ or ‘I ignore the rules completely’*). In a preliminary analysis we tested the construct validity the two COVID related questions (1) & (2) to assess emotional distress, finding high correlations with the BSI scores for somatization, depression, and anxiety.

**Table 2:** Socio-demographic distribution of the sample

Occupation						
Students	Employed	Free-lancers	retired	In training	unemployed	High school students/ unable to work
46%	30%	6%	9%	3%	3%	3%
Education						
University degree	High school diploma	PhD	Middle school			
46%	40%	7%	6%			
Living conditions						
With partner	Alone	Shared flat	With family			
31%	30%	22%	17%			
Living place (states of Germany)						
Berlin	Other					
51%	49%					

**Table 3:** Model Fit of all structural equation models

Model	χ^2^	*df*	*p*-value	CFI	RMSEA [CI]
Personality Functioning	3.67	4	0.452	1.00	0.000 [0.000, 0.092]
Personality Organization	129.00	58	< 0.001	0.943	0.070 [0.054, 0.086]
Defense mechanisms – adaptive defenses	2.64	2	0.267	0.999	0.036 [0.000, 0.136]
Defense mechanisms – intermediate defenses	6.95	2	0.031	0.990	0.099 [0.026, 0.184]
Defense mechanisms – maladaptive defenses	3.73	2	0.155	0.996	0.059 [0.000, 0.151]
Narcissism	8.54	4	0.074	0.991	0.067 [0.000, 0.130]
Psychological distress	10.29	15	0.801	1.00	0.000 [0.000, 0.039]

The model fit for the six models. Each model had one dimension of personality as predictor of the two variables “Emotional distress due to the challenges of the pandemic in general” and “Emotional distress related to the rules of social distancing

**Table 4:** Descriptive Statistics for the whole sample and divided by gender

	*General sample* *M (SD)*	*Female participants* *M (SD)*	*Male participants* *M (SD)*
Psychological Distress	1.73 (0.63)	1.73 (0.61)	1.70 (0.67)
PF	1.95 (0.54)	1.96 (0.51)	1.90 (0.58)
PO	2.05 (0.51)	2.04 (0.48)	2.05 (0.57)
Adaptive defensive mechanisms	4.44 (0.93)	4.40 (0.92)	4.57 (0.97)
Intermediate defensive mechanisms	4.52 (1.28)	4.57 (1.28)	4.30 (1.26)
Maladaptive defense mechanisms	3.05 (1.13)	3.02 (1.10)	3.09 (1.20)
Grandiose narcissism	3.21 (0.74)	3.19 (0.74)	3.24 (0.74)

For all scales, the mean values and corresponding SD over all items of the scales are shown. For Personality Functioning (LPFS-BF) the response scales ranged from 1 to 4. For Personality Organization (IPO) and psychological distress the response scales ranged from 1 to 5. For Defense Mechanisms (DSQ) the response scales ranged from 1 to 9. For Narcissism (PNI) the response scales ranged from 1 to 6.

**Table 5:** Means of different personality dimensions for different compliance groups

	‘I support the measures and comply with all rules’	‘I find the measures tend to be excessive, but still stick to them’	‘I make small exceptions now and then and expand the rules a bit if necessary’	‘I find the measures too rigid and bypass them if there are no consequences / it is not seen’ or ‘I ignore the measures completely’
PF	1.93 [1.84, 2.03]	1.93 [1.81, 2.06]	1.99 [1.83, 2.14]	2.37 [1.77, 2.98]
PO	1.96 [1.87, 2.05]	2.12 [2.01, 2.24]	2.15 [1.99, 2.30]	2.49 [1.71, 3.26]
Adaptive defense mechanisms	4.94 [4.75, 5.14]	5.12 [4.87, 5.37]	5.12 [4.76, 5.48]	4.77 [4.54, 5.00]
Intermediate defense mechanisms	4.51 [4.29, 4.73]	4.51 [4.17, 4.85]	4.59 [4.23, 4.96]	4.20 [2.96, 5.44]
Maladaptive defense mechanisms	2.92 [2.73, 3.12]	3.23 [2.98, 3.49]	3.10 [2.76, 3.44]	3.56 [1.94, 5.18]
Grandiose narcissism	3.19 [3.06, 3.32]	3.25 [3.08, 3.42]	3.24 [3.03, 3.46]	2.80 [2.04, 3.56]

For all scales, the mean values and corresponding confidence intervals over all items of the scales are shown. For Personality Functioning (LPFS-BF) the response scales ranged from 1 to 4. For Personality Organization (IPO-30) the response scales ranged from 1 to 5. For Defense Mechanisms (DSQ-40) the response scales ranged from 1 to 9. For Narcissism (PNI) the response scales ranged from 1 to 6

## Footnotes

1 As this study was part of a larger research project with multiple research questions, we changed order and numbering of hypotheses consecutively to increase the readability of the paper: H1 in this paper corresponds to H6b in the pre-registration; H2 in this paper corresponds to H6a in the pre-registration; H3 in this paper corresponds to H6c in the pre-registration. H4 in this paper is a non-preregistered exploratory research question that we added to our analysis after the pre-registration but prior to data analysis. We did not investigate on hypothesis 6d from the pre-registration as this research question is part of a collaborative data analysis independent from our current evaluation.

2 Germany had faced its first peak of the pandemic in April 2020 and been put under complete lockdown from March to May 2020. During the time of the study’s data collection, the lockdown was overcome, stores and restaurants had re-opened and the mandatory application of clinical face masks had just been introduced. Schools and universities were still closed, and a second wave of increasing infections and a second lockdown was expected to come. Vaccines had not been developed at this point.

3 Three individuals indicated that they completely ignored the restrictions and therefore were not experiencing any restrictions nor related emotional distress. These three individuals were excluded from further analyses.

4 Socio-demographic information on the sample is displayed in Table 2 in the Appendix.

## Declarations

### Human and animal research

The studies involving human participants were reviewed and approved by ethic commission (IRB) of the Psychologische Hochschule Berlin, Am Köllnischen Park 2, 10179 Berlin.
